# Class A CpG Oligonucleotide Priming Rescues Mice from Septic Shock *via* Activation of Platelet-Activating Factor Acetylhydrolase

**DOI:** 10.3389/fimmu.2017.01049

**Published:** 2017-08-30

**Authors:** Yoshinari Yamamoto, Ryu Sugimura, Takafumi Watanabe, Suguru Shigemori, Takuma Okajima, Shireen Nigar, Fu Namai, Takashi Sato, Tasuku Ogita, Takeshi Shimosato

**Affiliations:** ^1^Department of Bioscience and Food Production Science, Interdisciplinary Graduate School of Science and Technology, Shinshu University, Nagano, Japan; ^2^Research Fellow of the Japan Society for the Promotion of Science, Japan Society for the Promotion of Science, Tokyo, Japan; ^3^Department of Agricultural and Life Science, Graduate School of Science and Technology, Shinshu University, Nagano, Japan; ^4^Faculty of Medicine, Department of Intestinal Ecosystem Regulation, University of Tsukuba, Ibaraki, Japan; ^5^Metabologenomics Core, Transborder Medical Research Center, University of Tsukuba, Ibaraki, Japan; ^6^Department of Nutrition and Food Technology, Jessore University of Science and Technology, Jessore, Bangladesh; ^7^Department of Pulmonology, Graduate School of Medicine, Yokohama City University, Kanagawa, Japan; ^8^Department of Interdisciplinary Genome Sciences and Cell Metabolism, Institute for Biomedical Sciences, Shinshu University, Nagano, Japan; ^9^Department of Supramolecular Complexes, Research Center for Fungal and Microbial Dynamism, Shinshu University, Nagano, Japan

**Keywords:** Class A CpG oligodeoxynucleotide, sepsis, lipopolysaccharide, platelet-activating factor, platelet-activating factor acetylhydrolase, disseminated intravascular coagulation

## Abstract

Sepsis is a life-threatening, overwhelming immune response to infection with high morbidity and mortality. Inflammatory response and blood clotting are caused by sepsis, which induces serious organ damage and death from shock. As a mechanism of pathogenesis, platelet-activating factor (PAF) induces excessive inflammatory responses and blood clotting. In this study, we demonstrate that a Class A CpG oligodeoxynucleotide (CpG-A_1585_) strongly induced PAF acetylhydrolase, which generates lyso-PAF. CpG-A_1585_ rescued mice from acute lethal shock and decreased fibrin deposition, a hallmark of PAF-induced disseminated intravascular coagulation. Furthermore, CpG-A_1585_ improved endotoxin shock induced by lipopolysaccharide, which comprises the cell wall of Gram-negative bacteria and inhibits inflammatory responses induced by cytokines such as interleukin-6 and tumor necrosis factor-α. These results suggest that CpG-A_1585_ is a potential therapeutic target to prevent sepsis-related induction of PAF.

## Introduction

Toll-like receptors (TLRs) are a family of pattern recognition receptors distinguished by their role of phylaxis. TLRs recognize pathogen-associated molecular patterns (PAMPs) and activate immune signaling through innate and acquired immunity (1–3). Particularly, TLR4 recognizes lipopolysaccharide (LPS) derived from gram-negative bacteria and induces production of inflammatory cytokines such as tumor necrosis factor (TNF)-α, interleukin (IL)-1β, interferon (IFN)-γ, and IL-12 from macrophages and dendritic cells. However, excessive cytokine production results in a “cytokine storm,” which can induce lethal endotoxin shock (4, 5). TLR9 acts as a first-line host defense against pathogens recognizing DNA comprising unmethylated CpG motifs present in bacteria and viruses (6). Nucleic acid therapeutics including oligodeoxynucleotides (ODNs) from bacterial genomic DNA and microRNA are potential targeted therapies as they can strongly regulate gene expression and immune response (7–10). CpG motifs are at least 20-fold more common in bacterial DNA compared with vertebrate DNA and act as a PAMP (2). Mammalian TLR9 directly binds to unmethylated CpG DNA (CpG ODN) in the endolysosome/lysosome (11). CpG ODNs are classified into three main classes: A, B, and C. CpG ODNs have various immune functions according to their sequence (12). CpG ODN stimulates a strong innate immunity response, which may be inhibited by suppressive/inhibitory ODN (iODN) (7, 13). Several reports have described a protective effect for iODN. Interestingly, Shirota et al. reported that an iODN (A151) protects mice from endotoxin shock. In addition, A151 inhibited STAT1 and STAT4 phosphorylation and signaling cascade activated by IFN-β induced by LPS and IL-12. However, endotoxin shock worsened by treatment with Class B CpG ODN (5).

Various factors are associated with sepsis and the onset of endotoxin shock, as well as the induction of inflammatory cytokines. Platelet-activating factor (PAF) is a phospholipid that plays a significant role in inducing inflammation such as endotoxin shock and sepsis (14). PAF promotes platelet aggregation and activation and is involved in disseminated intravascular coagulation (DIC) and sepsis (15–17). Furthermore, PAF promotes the synthesis and release of immunological mediators such as TNF during inflammation (18, 19). Jacob et al. recently showed that PAF treatment induced acute lethality in mice (20). The circulating endogenous PAF level is controlled by PAF acetylhydrolase (PAF-AH), which is a phospholipase. In an LPS-induced lethal shock and cecal ligation and puncture (CLP) model, recombinant PAF-AH (rPAF-AH) improved the survival rate *via* suppressing inflammatory responses (21). PAF-AH and rPAF-AH were also demonstrated to improve the survival rate in a Phase II clinical study of patients with sepsis or multiple injuries (22). Therefore, PAF-AH is considered a therapeutic target for the treatment of sepsis and endotoxin shock. In this study, we examined the effect of a Class A CpG ODN 1585 (CpG-A_1585_) on coagulation and inflammatory responses to PAF-induced sepsis and LPS treatment. CpG-A_1585_ strongly induced PAF-AH, improved PAF-induced acute lethal shock and fibrin deposition, and rescued mice from LPS-induced endotoxin shock *via* inhibition of inflammatory responses. These results provide a new strategy against sepsis using Class A CpG ODN.

## Results

### CpG-A_1585_ Strongly Induces PAF-AH *via* TLR9

**Figure 1:**
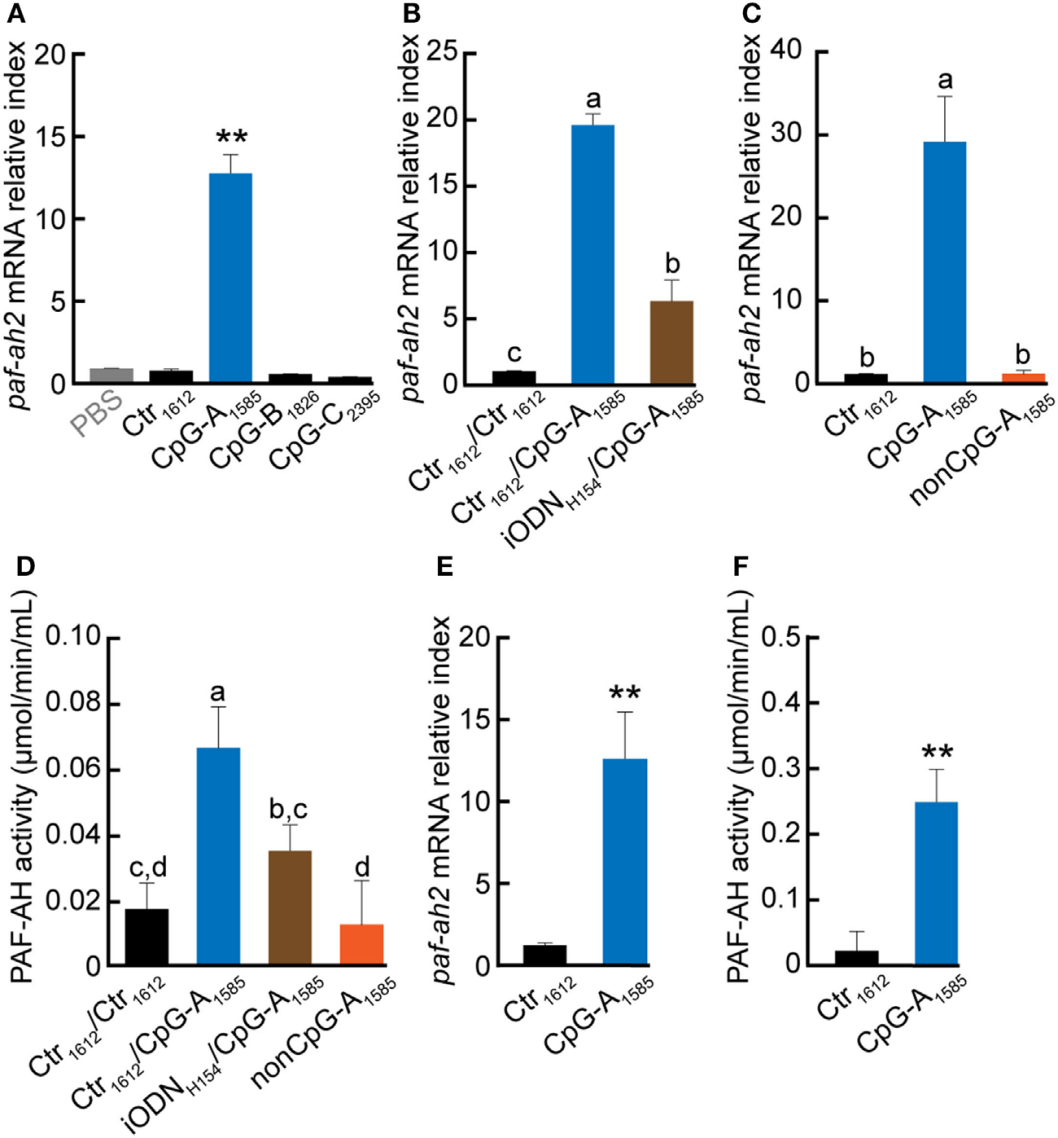
Effect of CpG oligodeoxynucleotide (ODN) on platelet-activating factor acetylhydrolase (PAF-AH) gene and enzymatic activity. Splenocytes or peritoneal macrophages were incubated with 3 µM CpG ODN, 3 µM CpG ODN 3 h after iODN_H154_ treatment, or non-CpG-A_1585_. After 24-h CpG ODN stimulation, the cells or supernatant were collected and used for PAF-AH expression or activity determination by quantitative PCR or PAF-AH assay kit, respectively. *Paf-ah2* mRNA expression by **(A)** CpG-A_1585_, CpG-B_1826_, and CpG-C_2395_; **(B)** iODN_H154_ inhibition; **(C)** non-CpG-A_1585_; and **(D)** PAF-AH activity in splenocytes. **(E)** *paf-ah2* mRNA expression and **(F)** PAF-AH activity in peritoneal macrophages. Data are presented as the mean ± SD. **p < 0.01, significant differences vs. phosphate-buffered saline and/or Ctr_1612_. Values with different letters (i.e., a, b, c, and d) represent significant differences (*p* < 0.05).

To investigate whether all classes of CpG ODN can induce PAF-AH, we examined the effect of CpG-A_1585_, -B_1826_, and -C_2395_ on induction of *paf-ah2* mRNA expression and PAF-AH activity in splenocytes *in vitro*. Interestingly, we found that only CpG-A_1585_ significantly induced *paf-ah2* mRNA expression compared to other ODNs (*p* < 0.01) (Figure 1A; Figure S1A in Supplementary Material). Inhibitory ODN H154 (iODN_H154_), a TLR9-specific antagonist, significantly suppressed CpG-A_1585_-induced *paf-ah2* mRNA expression and PAF-AH activity (*p* < 0.01) (Figure 1B). Since CpG-A_1585_ containing unmethylated CpG dinucleotides triggers the vertebrate immune response through TLR9 activation (23, 24), we examined whether a CpG motif in CpG-A_1585_ would affect CpG-A_1585_-induced *paf-ah2* mRNA expression using non-CpG ODN of CpG-A_1585_ (non-CpG-A_1585_). We found that Crt_1612_ and non-CpG-A_1585_ did not induce *paf-ah2* mRNA expression (Figure 1C). In addition, CpG-A_1585_ showed stronger PAF-AH activity compared with control (Ctr_1612_), whereas pretreatment of iODN_H154_ and non-CpG-A_1585_ showed insignificant PAF-AH activity compared with Ctr_1612_ (Figure 1D). Similar results were observed in peritoneal macrophages (Figures 1E,F). CpG ODNs, especially CpG-A, are known as IFN-α inducers (12). To investigate whether IFN-α is involved in induction of *paf-ah2* mRNA expression, we examined the response of polyinosinic-polycytidylic acid (poly(I:C)) as an IFN-α inducer and recombinant mouse IFN-α (rmIFN-α) on induction of *paf-ah2* mRNA expression. Poly (I:C) and rmIFN-α did not induce *paf-ah2* mRNA expression (Figures S1B,C in Supplementary Material). Taken together, these data suggest that only CpG-A_1585_ strongly induces PAF-AH *via* TLR9 but not IFN-α, and its production is derived from macrophages.

### CpG-A_1585_ Protects Mice from PAF-Induced Lethal Shock

**Figure 2:**
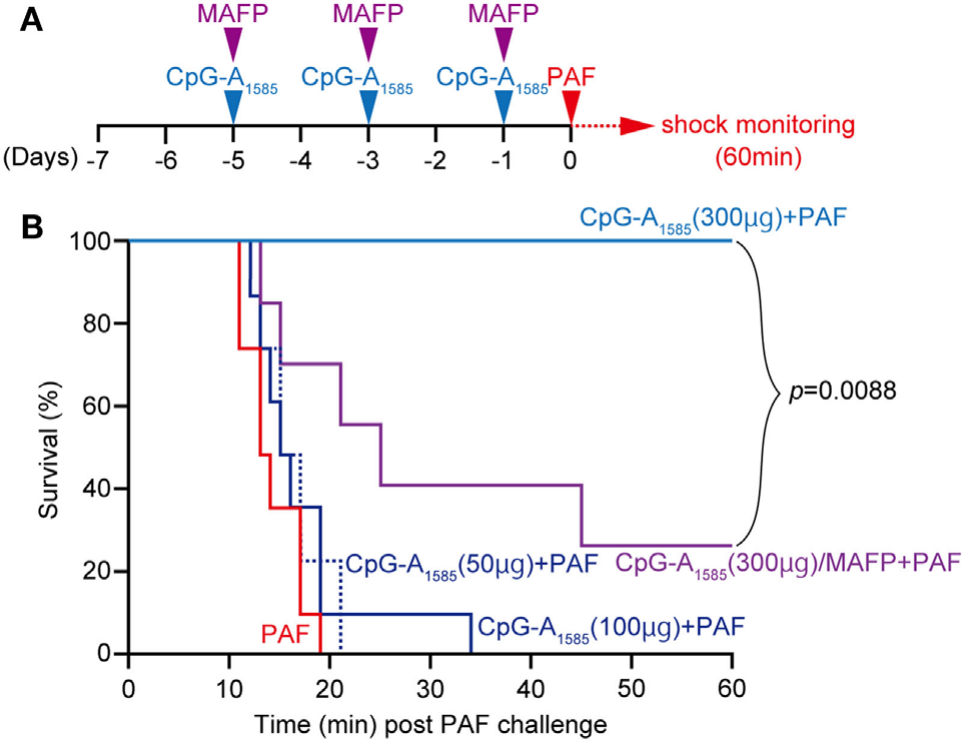
Protective effect of CpG-A_1585_ on platelet-activating factor (PAF)-induced lethal shock. **(A)** Experimental schedule to examine the effect of CpG-A_1585_ on PAF-induced sudden death. After 1 week of rearing, ICR mice were injected with 50, 100, or 300 µg CpG-A_1585_ with or without methyl arachidonyl fluorophosphonate (MAFP) (1 mg/kg) 1, 3, and 5 days before receiving 50 µg PAF containing 0.1% human serum albumin. **(B)** Survival was monitored for 60 min. *N* = 8 mice per group.

It has been reported that PAF administration alone induces sudden death in mice (20). Moreover, PAF is known to be involved in DIC as a symptom of sepsis (17, 25). Therefore, we examined the ability of CpG-A_1585_ to protect mice from PAF-induced lethal shock and sudden death. We administered 50, 100, and 300 µg CpG-A_1585_ intraperitoneally (i.p.) 1, 3, and 5 days before PAF challenge (Figure 2A). CpG-A_1585_ treatment at the highest dose (300 µg) significantly improved 20-min mortality in all mice compared with those in the PAF group (*p* < 0.0001), whereas 50 and 100 µg CpG-A_1585_ treatment did not advance mortality (Figure 2B).

We next applied methyl arachidonyl fluorophosphonate (MAFP) treatment, a PAF-AH inhibitor, to determine whether the effect of CpG-A_1585_ is dependent on PAF-AH. It has been shown that monocytes and thrombin-stimulated human coronary artery endothelial cells accumulated PAF following decreased PAF-AH when treated with MAFP *in vitro* (26, 27), and LPS-induced plasma PAF-AH activity was inhibited by MAFP *in vivo* (28). As expected, MAFP reduced the survival from 100 to 40% in the 300 µg CpG-A_1585_ group (Figure 2B). Taken together, CpG-A_1585_ protects mice from PAF-induced sudden death, and this effect is dependent on PAF-AH.

### CpG-A_1585_ Alleviates PAF-Induced Blood Clots

**Figure 3:**
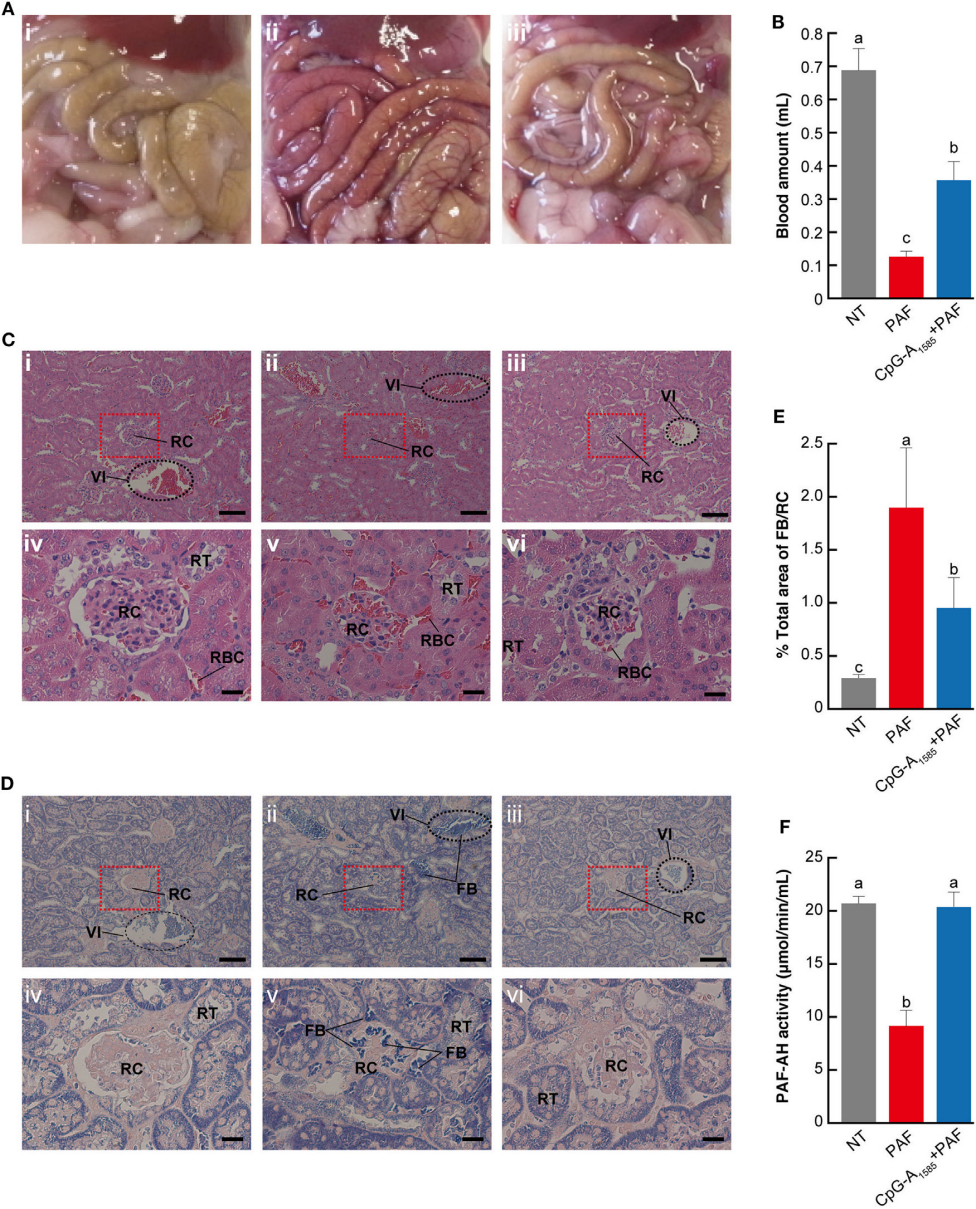
Mechanism of platelet-activating factor (PAF)-induced lethal shock ameliorated by CpG-A_1585_. ICR mice were injected with 300 µg CpG-A_1585_ for 1, 3, and 5 days before receiving 50 µg PAF containing 0.1% human serum albumin. Mice were then euthanized within 10 min after PAF injection. **(A)** Representative image of the abdominal cavity of (i) non-treated (NT), (ii) PAF-treated, and (iii) CpG-A_1585_ + PAF-treated mice. **(B)** Heart blood volume. **(C)** Hematoxylin-eosin staining, which shows polycythemia and morphological changes in the kidney. (i) NT, (ii) PAF-treated, and (iii) CpG-A_1585_ + PAF-treated mice. Scale bar = 100 μm. (iv) NT, (v) PAF-treated, and (vi) CpG-A_1585_ + PAF-treated mice. Scale bar = 20 μm. Fibrin thrombus is stained dark red. **(D)** Phosphotungstic acid hematoxylin staining, which shows fibrin accumulation in the kidney. (i) NT, (ii) PAF-treated, and (iii) CpG-A_1585_ + PAF-treated mice. Scale bar = 100 μm. (iv) NT, (v) PAF-treated, and (vi) CpG-A_1585_ + PAF-treated mice. Scale bar = 20 μm. Fibrin is stained dark blue. FB, fibrin; RBC, red blood cell; RC, renal corpuscle; RT, renal tubule; VI, interlobar veins. **(E)** The area of fibrin accumulation was determined by Image Processing Software, and the results are presented as the mean ± SE (*N* = 9 samples per group). **(F)** Serum platelet-activating factor acetylhydrolase (PAF-AH) activity in mice. *N* = 8 mice per group. Data are presented as the mean ± SE. Values with different letters (i.e., a, b, and c) represent significant differences (*p* < 0.05).

As our studies demonstrated that CpG-A_1585_ was effective for PAF-induced lethal shock, we sought to clarify the mechanism by which CpG-A_1585_ protects mice from PAF-induced lethal shock. Severe congestion was confirmed in the PAF group compared with the non-treated (NT) group, which was improved by CpG-A_1585_ treatment (Figure 3A). It was reported that PAF promotes platelet aggregation (15). Therefore, we investigated platelet aggregation by measuring heart blood volume. Heart blood volume in the PAF group was significantly decreased compared with the NT group (*p* < 0.01), whereas CpG-A_1585_ significantly improved heart blood volume (*p* < 0.01) (Figure 3B). In hematoxylin-eosin (HE) and phosphotungstic acid hematoxylin (PTAH)-stained sections (Figures 3C,D), increased fibrin in a fibrin thrombus and an interlobular vein to renal corpuscle was observed in the PAF group compared with the NT group. In the CpG-A_1585_ + PAF group, fibrin thrombus size and fibrinosis were significantly decreased compared with the PAF group (Figure 3E). Serum analysis showed that PAF-AH activity was significantly increased in the CpG-A_1585_ + PAF group compared with the PAF group (*p* < 0.01) (Figure 3F). These data suggest that CpG-A_1585_ ameliorates PAF-induced fibrin formation *via* PAF-AH activity.

### CpG-A_1585_ Inhibits TNF-α

**Figure 4:**
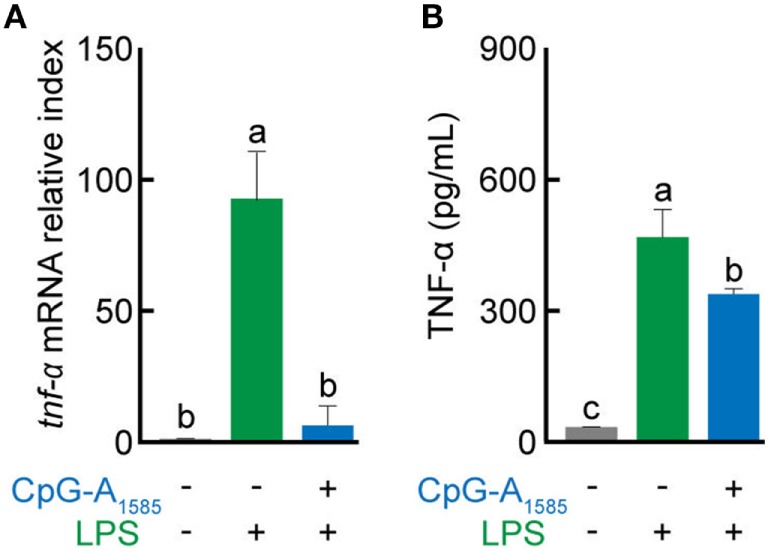
Suppressive effects of CpG-A_1585_ on lipopolysaccharide (LPS)-induced tumor necrosis factor (TNF)-α production. Splenocytes were first incubated with 3 µM CpG-A_1585_ and then incubated with LPS (10 ng/ml). After LPS stimulation, the cells or supernatant was collected and used to evaluate *tnf-*α mRNA expression or TNF-α protein level by quantitative PCR or enzyme-linked immunosorbent assay, respectively. **(A)** *tnf-*α mRNA expression and **(B)** TNF-α protein level in splenocytes. Data are presented as the mean ± SE. Values with different letters (i.e., a, b, and c) represent significant differences (*p* < 0.05).

Platelet-activating factor has been shown to play a fundamental role in the regulation of TNF secretion *in vitro* (29). Here, we examined the effects of CpG-A_1585_-induced PAF-AH on inflammatory responses generated by LPS stimulation. CpG-A_1585_ reduced LPS-induced TNF-α mRNA and protein expression levels (Figures 4A,B). These data suggest that CpG-A_1585_-induced PAF-AH can regulate inflammatory responses.

### CpG-A_1585_ Protects Mice from LPS-Induced Endotoxin Shock

**Figure 5:**
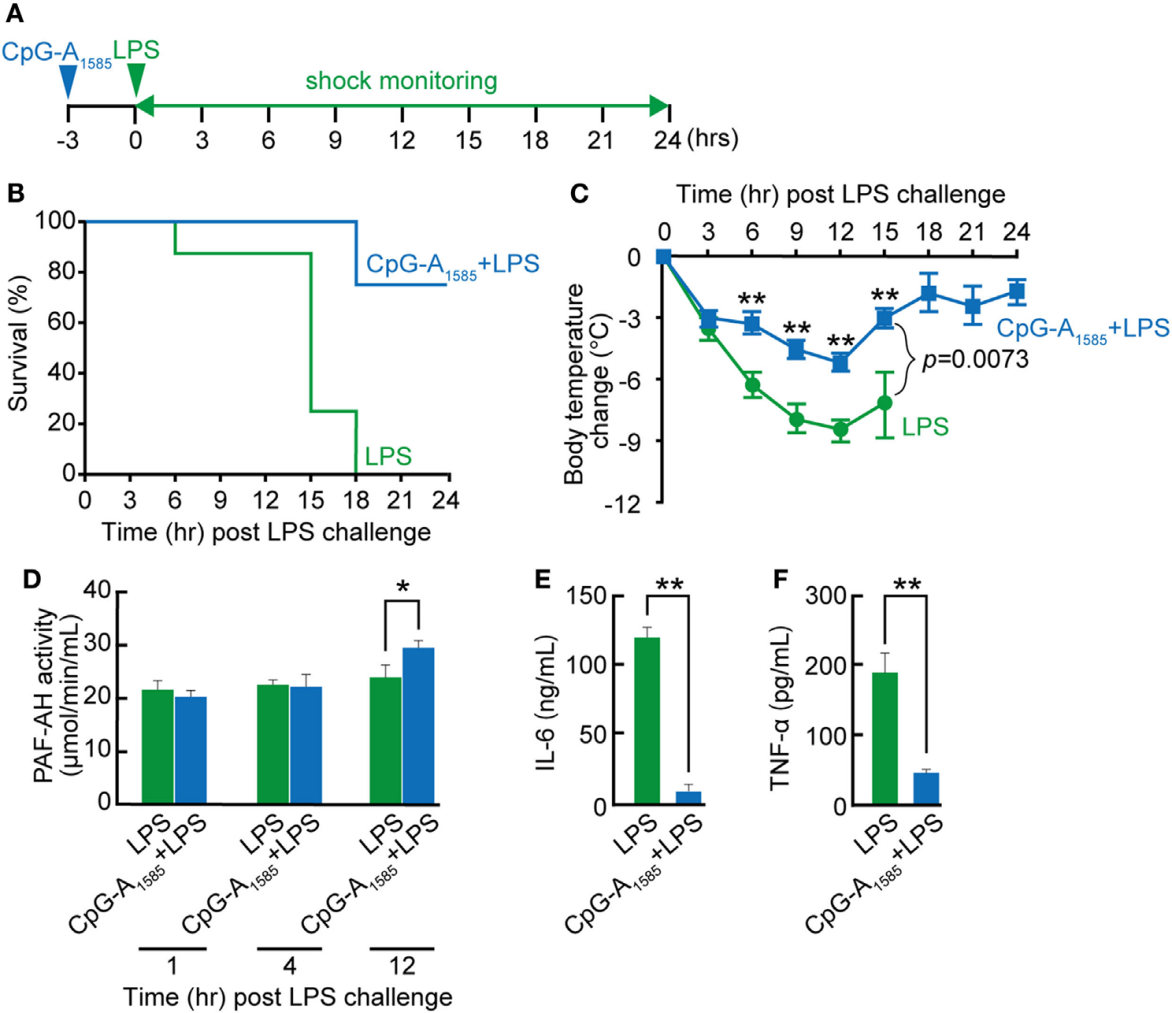
Protective effect of CpG-A_1585_ on lipopolysaccharide (LPS)-induced endotoxin shock. **(A)** Experimental schedule to examine the effect of CpG-A_1585_ on LPS-induced endotoxin shock. BALB/c mice were intraperitoneally injected with 300 µg CpG-A_1585_ 3 h before challenge with 750 µg LPS. **(B)** Survival and **(C)** changes in body temperature were monitored for 24 h. **(D)** Kinetics of platelet-activating factor acetylhydrolase (PAF-AH) activity in serum collected from the tail vein after receiving 300 µg CpG-A_1585_ and 750 µg LPS. **(E)** Serum interleukin (IL)-6 and **(F)** tumor necrosis factor (TNF)-α levels in mice that received LPS with or without 300 µg CpG-A_1585_ 12 h after LPS challenge. *N* = 8 mice per group. Data are presented as the mean ± SE. *p < 0.05 and **p < 0.01, significant differences between the LPS and CpG-A_1585_ + LPS groups.

In previous studies on the use of CpG ODNs for endotoxin shock and sepsis, Class B CpG ODN was confirmed to reduce survival in an experimental murine model of endotoxin shock or prevent sepsis-induced mortality (5, 30, 31). However, the effect of CpG-A ODN on endotoxin shock remains unclear. In addition, LPS is involved in PAF synthesis and PAF-induced diseases *in vitro* and *in vivo* (32, 33). Therefore, to determine whether CpG-A ODN could protect mice from LPS-induced endotoxin shock, mice were administered CpG-A_1585_ *via* i.p. injection 3 h before LPS challenge (Figure 5A). CpG-A_1585_ significantly improved 24-h mortality in the CpG-A_1585_ + LPS group compared with the LPS group (*p* < 0.0005) (Figure 5B). In addition, CpG-A_1585_ treatment improved hypothermia (Figure 5C). However, MAFP worsened hypothermia (Figure S2 in Supplementary Material). In addition, PAF-AH activity was significantly increased in the CpG-A_1585_ group compared with the control group at 12 h after LPS challenge (at 15 h after CpG-A_1585_ treatment) (*p* = 0.0199). IL-6 and TNF-α expression levels were significantly inhibited in the CpG-A_1585_ group compared with the control group at 12 h after LPS challenge (*p* = 0.0001 and *p* = 0.0001, respectively) (Figures 5D–F). Particularly, TNF-α expression levels in mice pretreated with CpG-A_1585_ (45.4 ± 17.6 pg/ml) were significantly lower than those in mice that received LPS only (190 ± 53.6 pg/ml) (*p* < 0.001). These results suggest that CpG-A_1585_ pretreatment effectively protects mice from LPS-induced endotoxin shock.

## Discussion

In 2016, the definitions of sepsis and septic shock were revised. Sepsis complicated by organ dysfunction was termed *severe sepsis*, which could progress to septic shock, defined as “sepsis-induced hypotension persisting despite adequate fluid resuscitation” (34). Sepsis is characterized by an excessive cytokine response and blood clotting reaction and is significantly involved in tissue injury and mortality (35). Here, we suggest that CpG-A_1585_ resolves septic and endotoxin shock through an anti-inflammatory response and improvement of DIC (Figures 2, 3 and 5).

**Figure 6:**
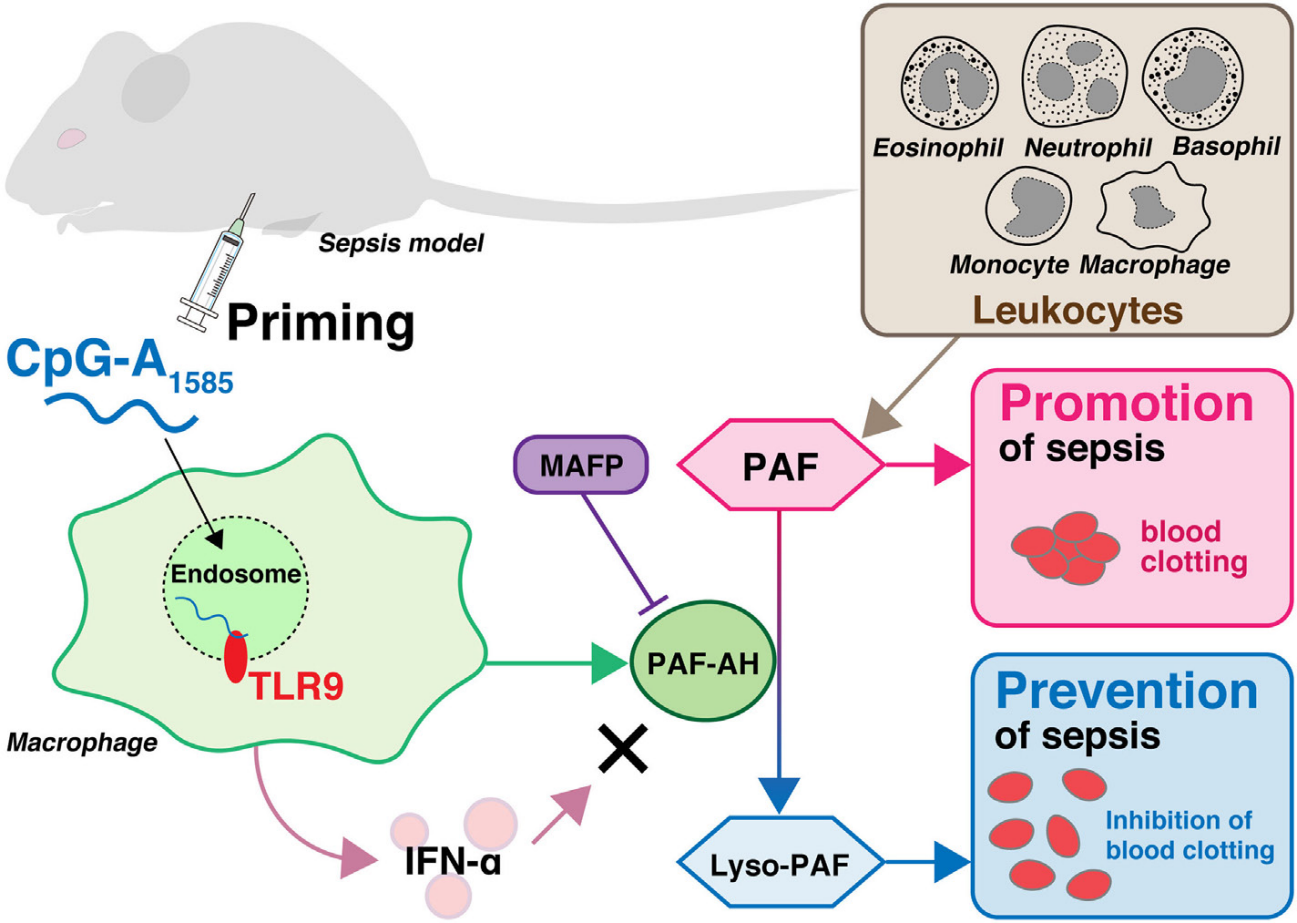
Overview of sepsis prevention by CpG-A_1585_ priming. Leukocytes including macrophages, monocytes, eosinophils, neutrophils, and basophils produce platelet-activating factor (PAF), which plays a significant role in the pathogenesis of endotoxin shock and sepsis in response to invading pathogens. PAF is degraded into lyso-PAF by platelet-activating factor acetylhydrolase (PAF-AH), resulting in the deactivation of PAF. CpG-A_1585_ induces PAF-AH from splenocytes, especially macrophages, *via* a TLR9-dependent pathway. Although CpG-A_1585_ is a strong inducer of interferon (IFN)-α, CpG-A_1585_-induced IFN-α does not affect PAF-AH. CpG-A_1585_ inhibits PAF-induced blood clotting *via* induction of PAF-AH, and this effect is inhibited by a PAF-AH-specific inhibitor, methyl arachidonyl fluorophosphonate (MAFP).

We showed that CpG-A_1585_ improved PAF-induced acute lethality, which was abrogated by a PAF-AH inhibitor, MAFP (Figures 2 and 6). Chen et al. showed that MAFP is a potent irreversible inhibitor of PAF-AH (26). In addition, Wu et al. also used MAFP as a specific inhibitor of PAF-AH and demonstrated that MAFP treatment inhibited plasma PAF-AH activity in an LPS-induced lung inflammation model. Further, Wu et al. mentioned that this finding strongly supported the involvement of PAF (28). In an experimental murine model of sepsis, acute lethal shock occurred by PAF-induced DIC. DIC is caused by pathological dysregulation of coagulation and fibrinolysis (36). The results of decreased heart blood volume and HE and PTAH staining demonstrated clear hallmarks of DIC (Figures 3B–D), particularly the presence of fibrin (25). CpG-A_1585_ treatment ameliorated blood loss and fibrin accumulation aggravated by PAF (Figures 3B–E) and significantly increased PAF-AH activity in serum (Figure 3F). In a previous study, the administration of a PAF antagonist improved DIC symptoms (17). This report suggests that PAF-inhibitors improve DIC. Namely, PAF-AH has the ability to improve DIC indirectly by generating lyso-PAF. Therefore, these results confirm that CpG-A_1585_ dissolves PAF *via* PAF-AH activation, improving PAF-induced sudden death in mice (Figure 6).

There are several experimental murine models of sepsis: (a) LPS-induced endotoxin shock model; (b) endogenic protection barrier model, such as CLP and colon ascendens stent peritonitis (CASP); and (c) external cause-related bacterial administration model. We used the LPS-induced endotoxin shock model, which was shown to exhibit systemic inflammation similar to initial clinical features in sepsis (37), and demonstrated that CpG-A improved endotoxin shock in this model. CpG-B was previously demonstrated to improve survival through IL-17 in the CLP model and enhanced cardiac dysfunction in the CLP model (30, 38). CpG-B may also stimulate neutrophil accumulation and improve survival by promoting bacterial exclusion (31). In another study, rPAF-AH administration increased bacterial clearance and enhanced the ability of macrophages to engulf invading bacteria (21). In addition, CpG-A_1585_ strongly induces PAF-AH, which may be useful for protection against CLP- and CASP-induced bacterial infection. Therefore, prevention and treatment of sepsis and control of inflammatory responses are important.

Various inflammation-associated factors, including LPS, TNF-α, IL-1, nitric monoxide, PAF, Braun’s lipoprotein (BLP), and high-mobility group box 1 protein, promote inflammation leading to sepsis (39, 40). It has been reported that anti-TNF antibody protects mice from LPS-induced lethal shock in a dose-dependent manner (41). Therefore, TNF-α control is a potential treatment strategy for sepsis and septic shock. In addition, an agonist of calcitonin gene-related peptide and pituitary adenylate cyclase-activating polypeptide type I receptor inhibited LPS-induced TNF-α and improved survival from endotoxin shock (42, 43). Our results supported these reports. In addition, BLP, a pro-inflammatory component of *Escherichia coli* membrane, stimulates endotoxemia similar to LPS *via* TLR2. It was also shown that PAF synthesis was induced by BLP (40). Taken together, CpG-A_1585_ may be effective for endotoxemia induced by both LPS and BLP.

In the present study, we further investigated CpG-A_1585_-induced PAF-AH using peritoneal macrophages and spleen cells (Figures 1D,F), as macrophages are known to secrete PAF-AH (44, 45). Moreover, several groups have reported TLR4/9 cross-tolerance (5, 46, 47), which is a strong anti-inflammatory LPS response induced by pretreatment with CpG-A_1585_ (47). Interestingly, our findings indicated that pretreatment with CpG-A_1585_ significantly increased PAF-AH in the early stage of TNF-α production in the septic/endotoxin shock model. In addition, we revealed that a PAF-AH inhibitor reduced survival in the PAF-induced acute lethal model. Hospitalized patients have a high risk of contracting septic shock because of weakened immune systems. In addition, sepsis is a disease that has low survival once contracted. CpG-A is an immunological enhancement molecule that is expected not only to prevent sepsis but also to enhance immune responses. Our findings confirm the importance of PAF and its function in septic/endotoxin shock.

## Materials and Methods

### ODNs and Reagents

Endotoxin-free desalted PS-ODNs were synthesized by Integrated DNA Technologies, Inc. (Coralville, IA, USA) or Gene Design, Inc. (Osaka, Japan). PS-ODNs were reconstituted in phosphate-buffered saline (PBS) and passed through a 0.22-µm pore microfilter (Nihon Millipore K.K., Tokyo, Japan). ODN sequences are shown in Table 1: CpG-A_1585_ (48), CpG-B_1826_ (49), CpG-C_2395_ (50), Ctr_1612_ (51), non-CpG-A_1585_, and iODN_H154_ (13). LPS from *Escherichia coli* 0127:B8 was purchased from Sigma-Aldrich (St. Louis, MO, USA). MAFP and PAF C-16 were purchased from Cayman Chemical Co. (Ann Arbor, MI, USA). Poly(I:C) was purchased from InvivoGen (San Diego, CA, USA). rmIFN-α was purchased from BioLegend (San Diego, CA, USA).

**Table 1:** ODN sequences.

Name	Sequence 5′–3′	Reference
CpG-A_1585_	G*GGGTCAACGTTGAG*G*G*G*G*G	(48)
non-CpG-A_1585_	G*GGGTCAAGCTTGAG*G*G*G*G*G	This study
CpG-B_1826_	T*C*C*A*T*G*A*C*G*T*T*C*C*T*G*A*C*G*T*T	(49)
CpG-C_2395_	T*C*G*T*C*G*T*T*T*T*C*G*G*C*G*C*G*C* G*C*C*G	(50)
Ctr_1612_	G*C*T*A*G*A*G*C*T*T*A*G*G*C*T	(51)
iODN_H154_	C*C*T*C*A*A*G*C*T*T*G*A*G*G*G*G	(13)

*Phosphorothioate bond.

### Mice

Female BALB/c and male ICR (Swiss albino) mice (6 weeks of age) were purchased from Japan SLC (Shizuoka, Japan), housed under temperature- and light-controlled conditions, and fed a standard diet (MF, Oriental Yeast Co. Ltd., Tokyo, Japan) and sterile water *ad libitum*. Mice were used for experiments after preliminary housing for 1 week.

### Cells and Cell Culture

Splenocytes from female BALB/c mice (8 weeks of age) were prepared using standard methods. Cells were seeded onto 24-well plates (Nalge Nunc International K.K., Tokyo, Japan) at a final concentration of 1 × 10^6^ cells/well in complete RPMI 1640 medium (Sigma-Aldrich) supplemented with 10% fetal calf serum (Sigma-Aldrich), 100 U/ml of penicillin, 100 mg/ml of streptomycin, 25 mM HEPES, 1.0 mM sodium pyruvate, non-essential amino acids, and 0.0035% 2-ME. Cells were then treated with 0.01–10 µM CpG ODN, 0.01–10 µg poly(I:C), or 0.01–1 ng rmIFN-α for 24 h (total 1 ml/well) (Figure 1; Figure S1 in Supplementary Material). After treatment, the cells and supernatant were collected for real-time quantitative PCR (qPCR) and PAF-AH enzymatic activity analysis, respectively. Cells were treated with 3 µM CpG-A_1585_ for 24 h, followed by stimulation with 10 ng/ml LPS for 6 h for qPCR analysis or for 24 h for enzyme-linked immunosorbent assay (ELISA) (Figure 4). Peritoneal macrophages were collected from mercy-killed female BALB/c mice (8 weeks of age) by peritoneal lavage with 5 ml of cold PBS, centrifuged at 1,500 rpm for 5 min and resuspended in medium. Murine peritoneal macrophages were then preincubated and adhered to dishes in medium for 24 h before exposure to CpG ODNs. Cells were seeded onto 24-well plates at a final concentration of 1 × 10^5^ cells/well and then treated with 3 µM CpG ODN for 24 h (total 1 ml/well) for qPCR analysis and PAF-AH enzymatic activity analysis (total 1 ml/well).

### PAF-Induced Lethal Shock Model

A schematic schedule of the experimental procedure is shown in Figure 2A. To determine the effect of PAF on the survival of ICR mice, we divided the animals (6 weeks of age) into the following three groups: PAF group, CpG-A_1585_ + PAF group, and CpG-A_1585_ + MAFP + PAF group (*N* = 8 mice per group). ICR mice were i.p. injected with 50, 100, or 300 µg CpG-A_1585_ 1, 3, and 5 days before PAF challenge. MAFP (1 mg/kg) was i.p. injected 20 min before CpG-A_1585_ injection. A stock solution of PAF was made in methanol, and the required aliquot was dried under a stream of nitrogen. PAF was then reconstituted in 0.5 ml PBS containing 0.1% human serum albumin (Wako Pure Chemical Industries, Ltd., Osaka, Japan) before use and administered i.p. into Swiss albino mice. After each treatment, animals were monitored for up to 60 min for survival.

### LPS-Induced Endotoxin Shock Model

A schematic schedule of the experimental procedure is shown in Figure 5A. BALB/c mice (7 weeks of age) were divided as follows: LPS group vs. CpG-A_1585_ + LPS group (Figure 5) and CpG-A_1585_ + LPS group vs. CpG-A_1585_/MAFP (5 mg/kg) + LPS group (Figure S2 in Supplementary Material) (*N* = 8 mice per group). BALB/c mice were i.p. injected with 300 µg CpG-A_1585_ 3 h before LPS challenge. Mice receiving MAFP (5 mg/kg) were i.p. injected 20 min before CpG-A_1585_ injection. The LPS-induced endotoxin shock model was established by i.p. injection of LPS (750 µg; Sigma-Aldrich). Survival was recorded at 0, 3, 6, 9, 12, 15, 18, 21, and 24 h. Body temperature (degree Celsius) was measured at 3-h intervals using an NTC thermistor (Tateyama Kagaku Industry Co., Ltd., Toyama, Japan).

### Blood Collection from Heart

Mice were euthanized by cervical dislocation 10 min after PAF injection. The maximum amount of blood was collected from the heart using a 10-ml syringe and 18 G needle within 5 min of death. The blood was weighed by ELECTRONIC BALANCE IBA-200 (AS ONE Corporation, Osaka, Japan). The volume (in milliliters) was calculated using the specific gravity (1.035) of mouse blood. The progression of blood clotting was evaluated by the quantity of collected blood.

### Histopathology

The kidney, liver (quadrate lobule), and spleen were fixed with 10% formalin neutral buffer solution (Wako Pure Chemical Industries, Ltd.), embedded in paraffin, sliced, and stained with HE and PTAH. Slicing and staining of embedded blocks were performed by Biopathology Institute (Oita, Japan). Histological pathology was evaluated under light microscopy. The area of fibrin accumulation was determined by Image Processing Software (Media Cybernetics Inc., Bethesda, MD, USA) and collected from three mice per group at adjacent axial locations.

### qPCR Analysis

Total RNA from the cells stimulated with CpG ODN and LPS was isolated using NucleoSpin^®^ RNA (TaKaRa Bio Inc., Tokyo, Japan). cDNA was prepared by reverse transcription from 1 µg of total RNA per sample using PrimeScript^®^ RT Master Mix (TaKaRa Bio Inc.). Equal volumes of cDNA were used for quantification of various cytokine cDNAs *via* qPCR using the Thermal Cycler Dice^®^ Real Time system (TaKaRa Bio, Inc.). qPCR analyses were performed with SYBR Premix Ex Taq (TaKaRa Bio, Inc.) using specific primers. Primers for β-actin, PAF-AH2, and TNF-α were purchased from TaKaRa Bio, Inc. For cross-sample comparison of results obtained following various treatments, cytokine mRNA levels were first normalized to those of β-actin mRNA. Data are shown as the mean ± SD of one representative experiment of three independent experiments with similar results.

### Enzyme-Linked Immunosorbent Assay

TNF-α level in sera or cell culture supernatants was quantified using a commercially available ELISA kit (TNF-α, eBioscience Inc., San Diego, CA, USA) according to the manufacturer’s instructions.

### PAF-AH Activity Assay

Platelet-activating factor acetylhydrolase activity in supernatant or sera was measured using the PAF-AH assay kit (Cayman Chemical Co.) (28). Briefly, 10 µl sample and 10 µl 5,5′-dithiobis (2-nitrobenzoic acid) were added to wells of a 96-well plate. The reactions were initiated by adding 200 µl substrate solution (2-thio-PAF). The absorbance at 405 nm was read every minute using a plate reader (iMark™ Microplate Reader, Bio-Rad, Hercules, CA, USA).

### Statistical Analysis

Statistical analyses were performed using a statistical software package (ystat2004.xls, Igaku Tosho Shuppan, Tokyo, Japan) or GraphPad Prism7 (GraphPad Software, Inc., La Jolla, CA, USA). All data were analyzed by one-way analysis of variance with the *post hoc* Student–Newman–Keuls test, except for survival analyses, *in vitro* qPCR analysis, PAF-AH activity assay, and all data from the LPS-induced endotoxin shock model. Survival analyses were performed using the log-rank test. *In vitro* qPCR analysis, PAF-AH activity assay, and all data from the LPS-induced endotoxin shock model were analyzed using Student’s *t*-test and the Dunnett test. Differences were considered significant at *p* < 0.05. Values for *in vitro* data are expressed as the mean ± SD. Other values are expressed as the mean ± SE.

## Ethics Statement

All experimental procedures were carried out in accordance with the Regulations for Animal Experimentation of Shinshu University, and the animal protocol was approved by the Committee for Animal Experiments of Shinshu University. Based on national regulations and guidelines according to Law No. 105 and Notification No. 6, all experimental procedures were reviewed by the Committee for Animal Experiments of Shinshu University (approval no. 280029).

## Author Contributions

YY, SN, TSA, TOG, and TSH conceived and designed the experiments; YY, RS, TW, SS, TOK, and FN conducted the experiments; YY and RS performed mathematical analyses; YY, SN, TSA, and TSH wrote the paper; TSH designed and supervised the work. All authors reviewed the manuscript.

## Conflict of Interest Statement

The authors declare that the research was conducted in the absence of any commercial or financial relationships that could be construed as a potential conflict of interest.

## Supplementary Material

Figure S1 Effect of CpG-A_1585_, CpG-B_1826_, CpG-C_2395_, and interferon (IFN)-α dose on platelet-activating factor acetylhydrolase (PAF-AH) mRNA expression. Splenocytes were incubated with 0.01–10 µM CpG oligodeoxynucleotide (ODN), 0.01–10 µg/ml poly(I:C), or 0.01–1 ng/ml rmIFN-α. After 24-h CpG ODN stimulation, the cells were collected and used for PAF-AH expression determination by quantitative PCR. *Paf-ah2* mRNA expression by **(A)** CpG-A_1585_, -B_1826_, and -C_2395_ dose, **(B)** poly(I:C) dose, and **(C)** rmIFN-α dose in splenocytes. Data are presented as the mean ± SD. ***p* < 0.01, significant differences compared to phosphate-buffered saline (PBS).

Figure S2 Effect of a platelet-activating factor acetylhydrolase inhibitor on amelioration of lipopolysaccharide (LPS)-induced endotoxin shock by CpG-A_1585_. **(A)** Experimental schedule to examine the effect of methyl arachidonyl fluorophosphonate (MAFP) on LPS-induced endotoxin shock ameliorated by CpG-A_1585_. BALB/c mice were intraperitoneally injected with 5 mg/kg MAFP for 20 min before receiving 300 µg CpG-A_1585_. After 3 h, the mice were challenged with 750 µg LPS. **(B)** Changes in body temperature within 24 h. *N* = 8 mice per group. Data are presented as the mean ± SE.
