# Supplementary figures and images for: Class A CpG Oligonucleotide Priming Rescues Mice from Septic Shock via Activation of Platelet-Activating Factor Acetylhydrolase
Source: Front Immunol. 2017 Aug 30;8:1049. doi: 10.3389/fimmu.2017.01049 (PMC5582170; doi:10.3389/fimmu.2017.01049)

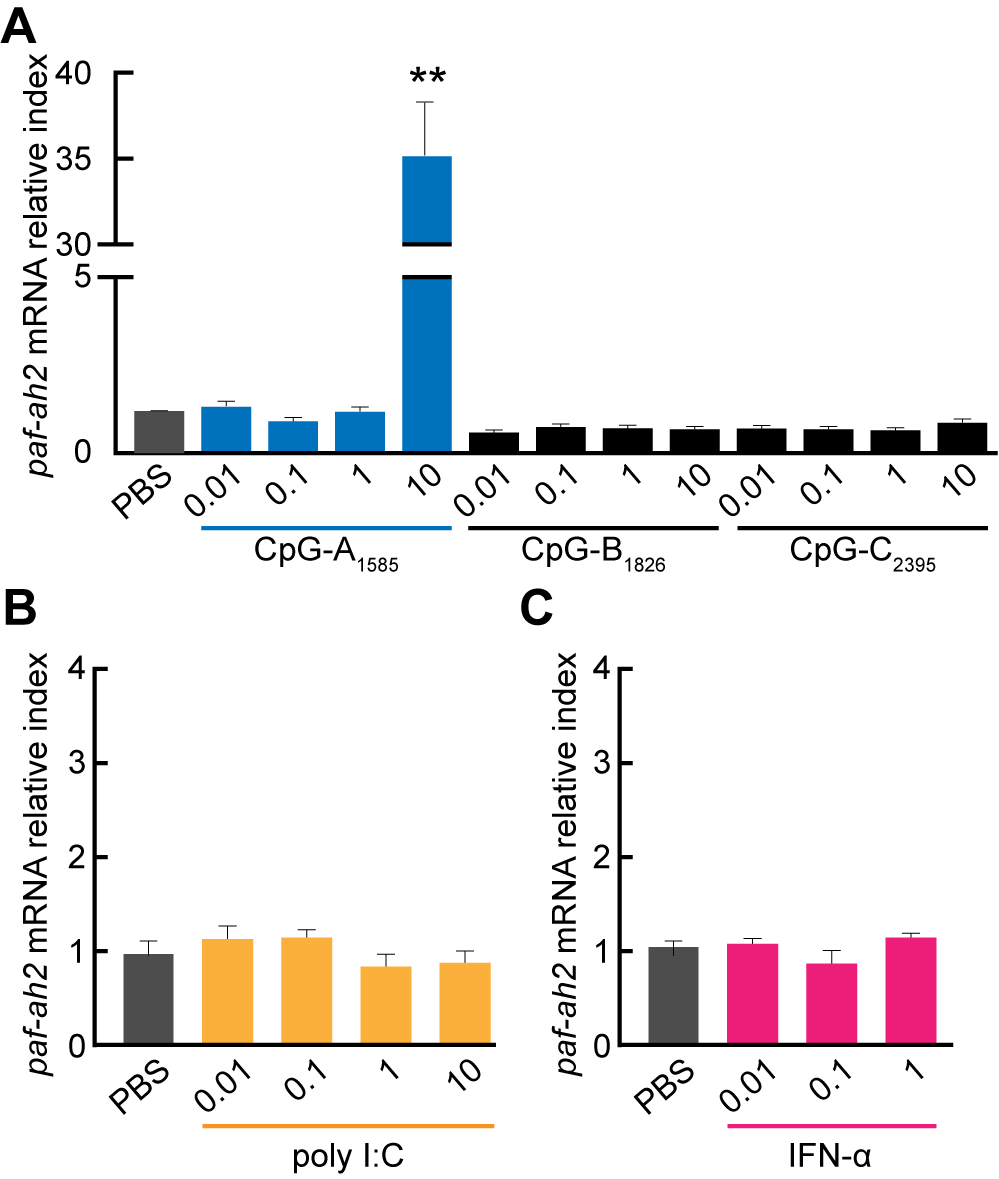

Supplement: Figure S1 — Effect of CpG-A1585, CpG-B1826, CpG-C2395, and interferon (IFN)-α dose on platelet-activating factor acetylhydrolase (PAF-AH) mRNA expression. Splenocytes were incubated with 0.01–10 µM CpG oligodeoxynucleotide (ODN), 0.01–10 µg/ml poly(I:C), or 0.01–1 ng/ml rmIFN-α. After 24-h CpG ODN stimulation, the cells were collected and used for PAF-AH expression determination by quantitative PCR. Paf-ah2 mRNA expression by (A) CpG-A1585, -B1826, and -C2395 dose, (B) poly(I:C) dose, and (C) rmIFN-α dose in splenocytes. Data are presented as the mean ± SD. **p < 0.01, significant differences compared to phosphate-buffered saline (PBS). [file image_1.tif]

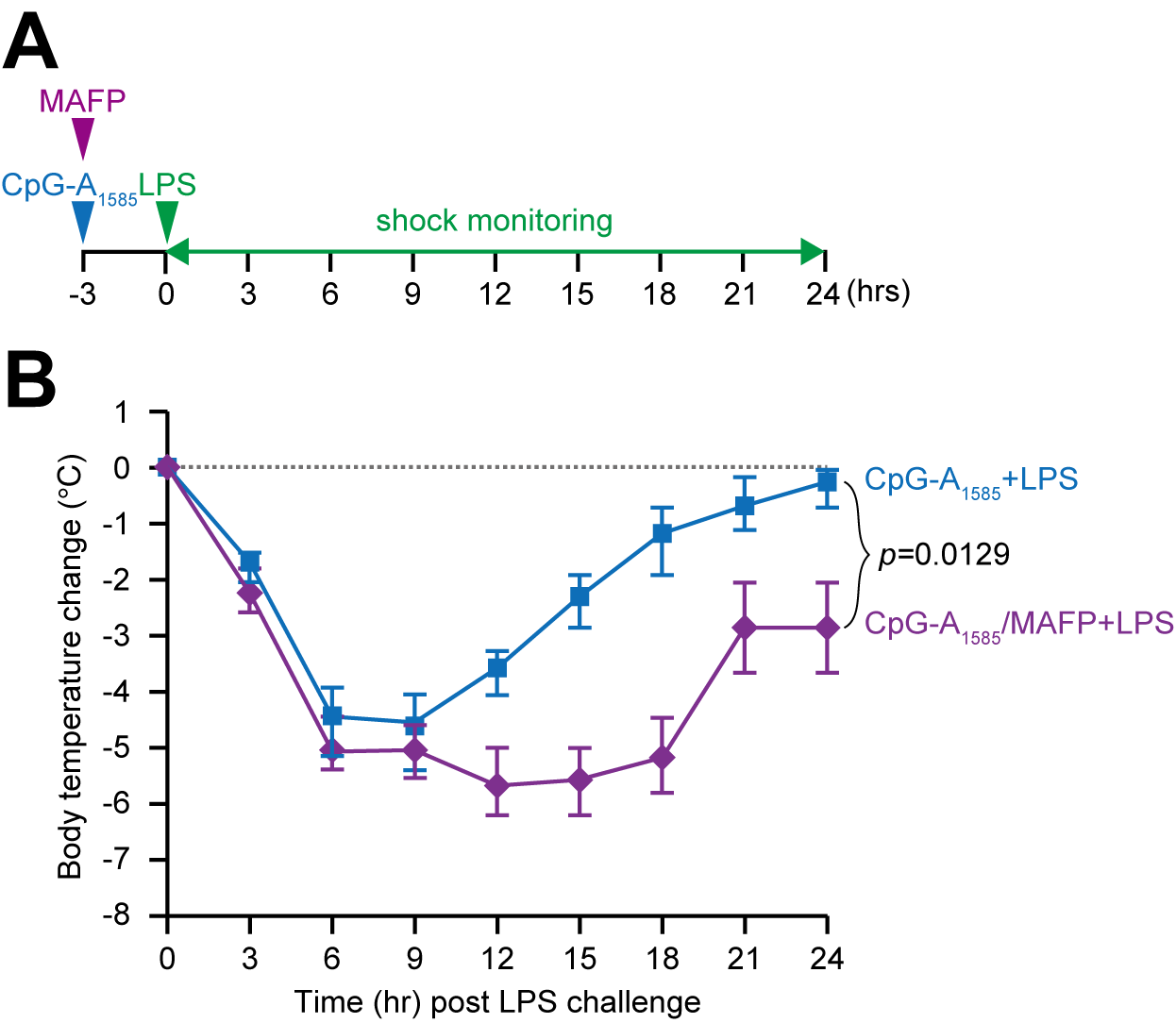

Supplement: Figure S2 — Effect of a platelet-activating factor acetylhydrolase inhibitor on amelioration of lipopolysaccharide (LPS)-induced endotoxin shock by CpG-A1585. (A) Experimental schedule to examine the effect of methyl arachidonyl fluorophosphonate (MAFP) on LPS-induced endotoxin shock ameliorated by CpG-A1585. BALB/c mice were intraperitoneally injected with 5 mg/kg MAFP for 20 min before receiving 300 µg CpG-A1585. After 3 h, the mice were challenged with 750 µg LPS. (B) Changes in body temperature within 24 h. N = 8 mice per group. Data are presented as the mean ± SE. [file image_2.tif]
